# Multistep non-fouling continuous flow synthesis and PEG-functionalisation of biocompatible iron oxide nanoparticles for magnetic hyperthermia, photothermal heating and antifungal activity

**DOI:** 10.1007/s41981-025-00355-2

**Published:** 2025-06-02

**Authors:** Sayan Pal, Georgios Gkogkos, Jacopo Piovesan, Zoe Whiteley, Maximilian O. Besenhard, Liudmyla Storozhuk, Martin R. Lees, Nguyen Thi Kim Thanh, Duncan Q. M. Craig, Alexander J. MacRobert, Sudaxshina Murdan, Asterios Gavriilidis

**Affiliations:** 1 Department of Chemical Engineering, University College London, Torrington Place, London, WC1E 7 JE UK; 2 Department of Biochemical Engineering, University College London, Bernard Katz Building, Gower St, London, WC1E 6BT UK; 3 UCL School of Pharmacy, University College London, 29-39 Brunswick Square, London, WC1N 1AX UK; 4 UCL Healthcare Biomagnetics Laboratories, University College London, 21 Albemarle Street, London, W1S 4BS UK; 5 UCL Nanomaterials Laboratory, University College London, 21 Albemarle Street, London, W1S 4BS UK; 6 Biophysics Group, Department of Physics and Astronomy, University College London, Gower Street, London, WC1E 6BT UK; 7 Department of Physics, University of Warwick, Coventry, CV4 7AL UK; 8 Department of Surgical Biotechnology, University College London, Royal Free Campus, London, NW3 2PS UK; 9 Faculty of Science, University of Bath, Claverton Down, Bath, BA2 7AY UK

**Keywords:** Millifluidic reactor, On-line functionalisation, Iron oxide nanoparticles, Magnetic hyperthermia, Photothermal effect, Antifungal activity

## Abstract

An innovative method for synthesising and functionalising iron oxide nanoparticles (IONPs) with polyethylene glycol (PEG) using a continuous three-phase segmented flow reactor is presented. Integration of synthesis and functionalisation within a single reactor platform eliminates the need for laborious batch post-processing steps, such as washing, separation, and dialysis, significantly reducing processing time and enhancing efficiency. The incorporation of oleic acid during the PEG functionalisation step further improved colloidal stability, resulting in 15 nm nanoparticles that remained stable for months without precipitation. FTIR and TGA confirmed successful functionalisation, while XRD showed the absence of byproducts. The PEG-functionalised IONPs exhibited excellent biocompatibility, as confirmed by in vitro cytotoxicity assays, with cell viability exceeding 80% at biologically relevant concentrations. Importantly, the functionalisation process preserved the nanoparticles’ key magnetic and thermal properties, such as saturation magnetisation, magnetic heating efficiency and photothermal response, which are essential for their application in therapeutic settings. Biomedical applications of these functionalised IONPs were explored across multiple domains. The nanoparticles showed efficient magnetic hyperthermia performance under an alternating magnetic field, making them suitable for cancer treatment via localised heating. Additionally, their photothermal properties were assessed under near-infrared (NIR) irradiation, demonstrating temperature rise proportional to concentration, and hence their potential for dual-mode therapeutic applications. Furthermore, antifungal activity assays revealed PEG-functionalised IONP’s efficacy against *Trichophyton rubrum*, with complete fungal growth inhibition at specific concentrations, underscoring their potential in pharmaceutical antifungal formulations. The continuous flow process developed offers a robust platform for producing multifunctional nanoparticles tailored for biomedical applications, while ensuring compatibility with industrial-scale production demands.

## Introduction

Significant advances in the functionalisation of nanoparticles are important for their application in pharmaceutical and biomedical sciences. Preliminary clinical findings indicate that functionalising nanoparticles with specific recognition chemical moieties can produce multifunctional nanoparticles that exhibit increased efficacy while simultaneously minimising side effects [1, 2]. Among the diverse classes of nanoparticles, iron oxide nanoparticles (IONPs) have been the focus of extensive research due to their broad range of applications in the biomedicine and healthcare sectors. While continuous flow synthesis of IONPs using various synthetic methods has been reported over the past decade [3–6], the functionalisation of IONPs is typically conducted through a time-consuming batch process. The functionalisation step of nanoparticles in batch processing is a significant bottleneck in the manufacturing workflow, hindering the realisation of key advantages of continuous nanoparticle synthesis, including scalability, consistent quality, and cost efficiency.

Magnetic IONPs are widely reported for a wide range of biomedical applications, such as magnetic heating [7] and photothermal heating [8, 9] for cancer treatment, as well as pharmaceutical applications such as antifungal agents [10]. Recent reports have shown that heating efficiency was enhanced using magnetic and photothermal heating effects together at biologically safe limits [11], while other reports have shown IONPs to be effective against fungal infections by various fungal species [10, 12, 13]. An aqueous coprecipitation-based continuous flow synthetic method of IONPs with good magnetic heating performance was reported in our recent work using a seeded growth technique in a three-phase segmented flow reactor. However, it is reported that the biocompatibility of IONPs synthesised by co-precipitation method is not satisfactory [14]. For in vivo use of the IONPs, or their use in antifungal formulations, the nanoparticles also need to exhibit good biocompatibility. Various researchers reported functionalisation/coating of IONPs with polyethylene glycol (PEG), which is an FDA-approved excipient and can be used to alter their reactive surface, significantly improving their biocompatibility [14–17]. PEG is known to enhance steric colloidal stabilisation by reducing nanoparticle interactions through increased steric hindrance. Additionally, the ether repeat units in PEG promote hydrophilicity by forming hydrogen bonds with the surrounding solvent, further improving nanoparticle dispersion [18]. In recent years, PEG, PEG mixed with weak acids, activated PEG-weak acid complexes (such as folic acid and PEG-diacids [19, 20]), are reported for effective PEG functionalisation of IONPs. However, these functionalisations are usually performed in conventional batch processes, require separation, washing and dialysis steps and involve very long steps in the order of hours to days. PEG functionalisation of IONPs in continuous flow has been uncommon and the only relevant report by Mahin et al. [19] focuses on tetraethylammonium hydroxide-based coprecipitation chemistry, which is inherently non-fouling and leads to small-sized IONPs (< 10 nm) not suitable for magnetic hyperthermia. However, in numerous synthetic methods for IONP production, challenges such as fouling and difficulties in continuous flow operation exist.

The current study aims to address the challenges associated with the online PEG functionalisation of IONPs by developing a continuous-flow synthesis process followed by PEG functionalisation in the presence of oleic acid, which is additionally reported to enhance colloidal stability and minimize nanoparticle agglomeration [21]. Seeded-growth coprecipitation synthesis of IONPs was performed, followed by online PEG functionalisation within a three-phase segmented flow reactor developed in our previous work [4, 22]. This approach eliminates the need for additional workup steps and significantly reduces overall processing time. Such a reactor can be used to prevent clogging (that may arise during IONP formation) and to provide reaction mixture compartmentalisation, fast mixing characteristics and to enable precisely timed reagent addition. The PEG-coated IONPs synthesised demonstrated good biocompatibility and potential for magnetic hyperthermia, photothermal heating and antifungal formulations.

## Experimental

### Materials

Iron (III) chloride hexahydrate (99%, Sigma Aldrich), iron (II) chloride tetrahydrate (99%, Sigma Aldrich), sodium carbonate decahydrate (99%, Sigma Aldrich), citric acid (CA) (99%, Sigma Aldrich), oleic acid (OA) (technical grade), hydrochloric acid (1 M, VWR), polyethylene glycol (PEG-6000, VWR), heptane (99%, VWR) and nitrogen gas (zero grade) were used as received without any further purification. All solutions were prepared freshly before each experiment using deionised water (DI, 15 mΩ). IONPs were synthesised via the seeded growth coprecipitation synthetic method. Iron precursor solution of total iron concentration of 0.1 M was prepared by mixing 1.802 g iron (III) chloride hexahydrate and 0.6627 g iron (II) chloride tetrahydrate in 100 mL of deionised water, keeping the [Fe^3+^]/[Fe^2+^] molar ratio as 2. Sodium carbonate base solution (0.33 M) was used for reduction. After synthesis of the IONPs, different solutions were added for colloidal stability, such as 0.31 M citric acid solution, 10 w/v % solution of PEG-6000 and 10 w/v % solution of PEG-6000 with 0.08 mM oleic acid. All reaction solutions were deoxygenated via N_2_ bubbling for at least 30 min before the reaction. The PEG-oleic acid solution was sonicated for 5 min before the deoxygenation step. The reactor set-up was cleaned with isopropyl alcohol (VWR) and 1 M HCl solution between experiments to prevent any contamination.

### Nanoparticle characterisation

The core diameter (*D*_*TEM*_) of the synthesised IONPs was determined from transmission electron microscopy (TEM) images captured at 120 kV acceleration voltage (JEOL 1200 EX). Average particle size was obtained by randomly selecting ≥ 100 individual particles on different TEM images and measuring their size using ImageJ 1.8. After collection of the two phase (aqueous-organic) sample from the reactor, the organic phase was removed by decantation and IONPs were obtained from their aqueous solution by centrifugation (Allegra 64R, Beckman Coulter) followed by re-dispersion in DI water via ultra-sonication. To prepare TEM Grids, drops of this washed sample solution were placed on a carbon coated copper grid (200 $\mu$m lattice). The hydrodynamic diameter, *D*_*h*_, of the IONPs was measured using dynamic light scattering (DLS) (DelsaMax Pro, Beckman Coulter). The concentration of Fe in the nanoparticle solution was measured via microwave plasma atomic emission spectroscopy (MP-AES, Agilent 4210) as described in our previous work [3]. A Perkin Elmer TGA 7 thermogravimetric analyser was used for thermogravimetric analysis of washed and dried (by evaporation at room temperature) IONP samples and Fourier transform infrared (FTIR) spectra were recorded using an attenuated total reflectance probe (Spectrum 100 FTIR, Perkin Elmer). Magnetisation hysteresis curves were determined via a superconducting quantum interference device (SQUID) magnetometer (MPMS, Quantum Design) at 5 K and 300 K. Washed IONP solutions were dried by evaporation at room temperature and loaded in a capsule adapted for SQUID measurements.

The nanoparticles’ heating abilities in an alternating magnetic field were characterised with a G2 driver D5 series calorimetric *analyser* from nB nanoScale Biomagnetics at a magnetic field strength of 23.87 kA/m and a frequency of 488 kHz. The IONPs’ specific absorption rate (SAR) and the intrinsic loss power (ILP) [23] were used to compare heating rates*,* and were determined as described previously [24]*.*

The photothermal assessment of the PEG-coated IONPs was performed using a near infrared light emitting diode (NIR-LED, Thorlabs M810L5) as light source, emitting at a wavelength of 810 nm at 420 mW (medium setting – measured at 2 mm away from the light source). For the measurement, a 3D printed sample holder was used as in our previous work [25]. A sample (2 mL) was loaded in a quartz cuvette (Hellma) of 10 mm optical path with opaque side walls. The cuvette was held in place using the 3D printed holder on top of a magnetic stirrer. A small 3 mm × 9 mm cylindrical stir bar was placed in the cuvette and all measurements took place under stirring at 500 rpm. A thermocouple, connected to a data logger (TC-08, Pico Technology), was placed in contact with the side wall of the cuvette, held by the 3D printed holder. A piece of reflective aluminium foil was placed around the opaque sides of the cuvette (including between the cuvette and the thermocouple) to prevent direct illumination of the thermocouple by the diverging NIR-LED beam and reduce the effects of direct heating of the holder surfaces in contact with the cuvette. The temperature was logged using PicoLog™ software every 0.1 s (logging started the moment the NIR-LED was turned on). The concentration was controlled by diluting the IONP sample with DI water.

Antifungal susceptibility investigations were performed by a broth microdilution approach. The method implemented was a modification of the CLSI (M27-yeast & M38-dermatphyte) and EUCAST (7.3.2-yeast & 11.0-dermatophytes). The absorbance of the plates was recorded with BioTek 800 TS microplate reader at 630 nm. Each experiment was performed 3 times, and their absorbance was averaged. The absorbance of the nanoparticle suspension was subtracted from the total absorbance to obtain a plot indicating the percentage of fungal growth at various IONP concentrations. *Trichophyton rubrum* (ATCC28188) was grown for 7 d at 35 °C on SDA dishes. 5–10 mL of 0.9% saline solution was added to the plate to prepare a suspension which was then filtered to remove hyphal fragments and micro-conidia. The final suspension was adjusted spectroscopically to match the absorbance of McFarland 0.5 standard (2–5 × 10^6^ CFU/mL). The IONP samples were loaded in a flat-bottom 96 well plate using a two-fold dilution approach. The stock inoculum suspension was first diluted 1:50 with RPMI-1640 and then added to each well to obtain a final inoculum concentration of 1–5 × 10^4^ CFU/mL. The absorbance of the plate was collected upon preparation to account for the absorbance arising from the nanoparticle suspension at the highest concentrations. The plates were incubated at 35 °C for one week. The results were analysed visually and spectroscopically.

Cytotoxicity of the IONPs was assessed using an MTT (3-(4, 5-dimethylthiazolyl-2)−2, 5-diphenyltetrazolium bromide) assay, measuring cell viability and proliferation. The assay used was the CellTiter 96® non-radioactive cell proliferation assay kit (Promega, UK), performed on SH-SY5Y cells. For the SH-SY5Y cells, a growth medium mixture of 1:1 Eagles’ Minimum Essential Medium (EMEM) and F12 medium were used, with the addition of 10% fetal bovine serum (FBS) and 1% penicillin–streptomycin. All cell culture media and components were purchased from Gibco (Loughborough, UK). The cells were plated in 96-well plates at a seeding density of 0.1 $\times$ 10^5^ cells/well with 100 µL of media, where they were incubated for 24 h at 37 °C and 5% CO_2_. Upon reaching 80–90% confluency, the diluted IONP solutions were added to the cells in triplicate. The washed IONP stock solutions were diluted to form the following final concentrations upon addition to the wells; 27.63 *µ*g/mL (100 X), 110.52 *µ*g/mL (25 X) and 921 *µ*g/mL (3 X). After the addition of particles to the wells and incubation for 24 h, the culture media was aspirated and replaced with a mixture of 100 µL media/well and 15 µL MTT dye/well and the plate was incubated at 37 °C and 5% CO_2_. After 1 h of incubation, 100 µL of MTT stop solution was added to each well and the plate incubated for 24 h at 4 °C. The absorbance of each well was measured at 570 nm using a SpectraMax microplate reader (Molecular Devices, UK) and cell viability was consequently calculated: $\%Cell\,Viability= \frac{\left[Abs\right]nanoparticle}{\left[Abs\right]control}\times 100$, where *[Abs]nanoparticle* is the absorbance for the wells containing nanoparticles and *[Abs]control* is the absorbance for wells containing cells and media only, as the negative control. The results represent the mean ± standard deviation of 3 independent MTT assays with 3 repeats of all samples each time.

### Synthesis of functionalised IONPs using a three-phase flow reactor

**Fig. 1:**
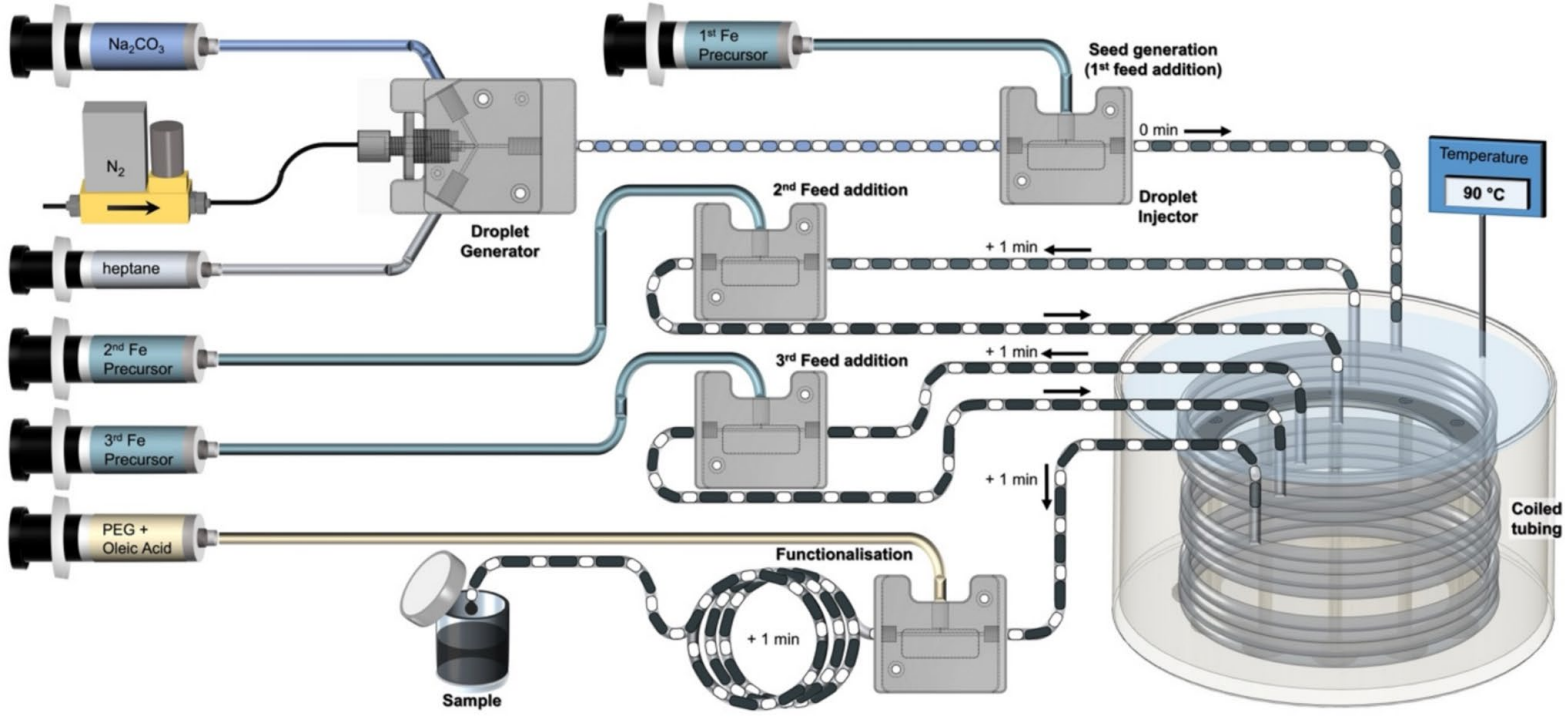
Schematic of experimental setup for continuous flow co-precipitation IONP synthesis with online functionalisation. The figure corresponds to functionalisation with PEG-oleic acid

A three-phase flow reactor (see Fig. 1) developed in our previous work was used for the continuous flow synthesis of functionalised IONPs [4, 22]. Five syringe pumps (Harvard Apparatus, PHD Ultra), equipped with six 50 mL gas-tight syringes, were employed to independently regulate the input streams of the reactant (Fe precursor solution and Na_2_CO_3_ solution), carrier phase (heptane) and functionalisation solutions (CA, PEG, PEG-OA solutions) into the reactor. A custom-made droplet generator was used to form liquid–gas-liquid (heptane-nitrogen-aqueous base) three phase flow in the reactor. Nitrogen gas was fed to the droplet generator at atmospheric pressure via a mass flow controller (0.1—5 mL/min EL-FLOW Prestige, Bronkhorst) as the inert gas phase. After formation of three phase flow in the droplet generator, precursor solutions were injected into the aqueous phase droplets in three steps using custom designed droplet injectors. After the IONP synthesis finished, for the online functionalisation step, CA solution, or PEG solution or PEG-OA solution was injected into the aqueous droplets using a fourth droplet injector. Coiled PTFE tubing (1 mm diameter) submerged in a temperature bath kept at 90 °C was used between each injection steps to provide 1 min residence time at desired reaction temperature for each step. Sodium carbonate solution, nitrogen, heptane, iron precursor solution, CA and PEG/PEG-OA functionalisation solution flow rates were kept constant at 0.4, 0.4, 0.2, 0.133, 0.168 and 0.02 mL/min respectively. The reactive aqueous (dispersed) phase was compartmentalised and isolated from the reactor walls by the continuous phase. The aqueous chemistry taking place within plastic capillaries was rendered non-fouling by employing a non-polar liquid (heptane) and gas phase, thereby preventing interactions with the reactor walls. A detailed discussion on these aspects of the flow reactor platform, operating procedure and three-phase flow dynamics are reported in our previous work [4].

## Results and discussion

### Particle functionalisation, stability and physical characterisation

**Fig. 2:**
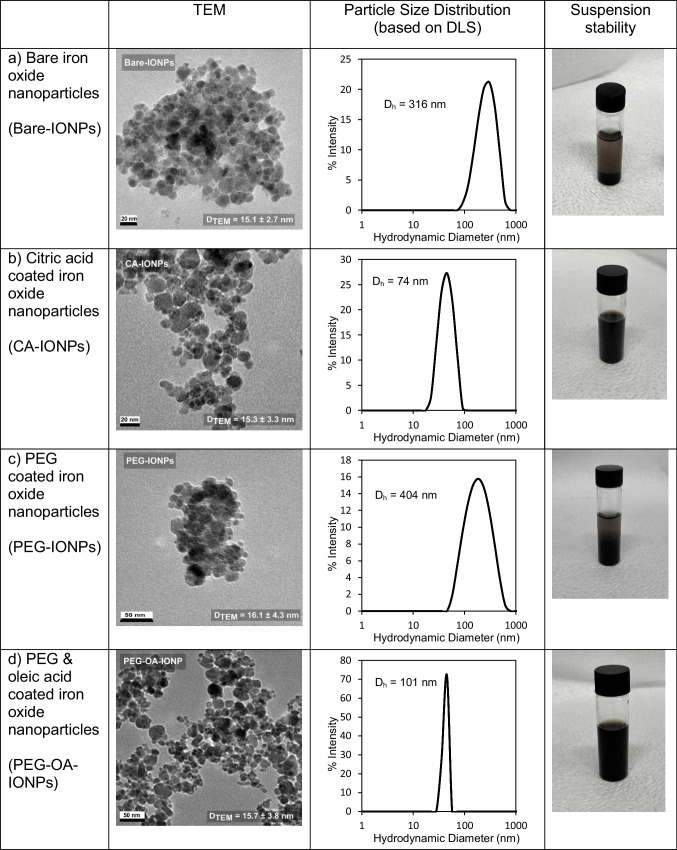
Representative TEM images with *D*_*TEM*_ and standard deviation, distributions of hydrodynamic diameter, *D*_*h*_, and suspension stability of IONPs following functionalisation with different additives. (**a**) Bare-IONPs, (**b**) CA-IONPs, (**c**) PEG-IONPs, (**d**) PEG-OA-IONPs

IONP seeds were formed in the first iron precursor feeding step of the three-phase flow reactor, followed by two subsequent feeding steps where more iron precursor solution was added into the reacting droplets, enabling seeded growth. Figure 2 shows the particle sizes obtained by TEM and DLS, along with pictures indicating the stability of the samples. It is important to note that while monodisperse and non-agglomerated particles typically yield comparable average sizes when measured by TEM and DLS, the presence of agglomeration and polydispersity precludes such comparison between TEM and DLS-derived particle sizes in this study. The IONPs collected after the synthesis steps without any functionalisation (bare-IONPs) were found to be unstable and precipitated in the collection vial after a few minutes. CA, PEG and PEG-OA solutions were added to already grown bare-IONPs at the 4th droplet injector for deagglomeration/functionalisation of the IONP suspension. CA-IONPs were found to be colloidally stable without forming any precipitate (see Fig. 2). However, the IONPs were not colloidally stable with only PEG solution addition (PEG-IONPs) and formed agglomerates. The hydrodynamic diameter (*D*_*h*_) of the PEG-IONPs was significantly higher (404 nm) than colloidally stable samples (~ 70–100 nm). The addition of OA with PEG was found to aid the stabilisation process of the IONPs, resulting in lower *D*_*h*_ (from 404 to 101 nm). The antifouling three phase flow reactor was capable of synthesising IONPs with only PEG solution, without any reactor fouling or clogging despite excessive aggregate formation. However, PEG-IONPs formed a precipitate in the collection vial after a few minutes (see Fig. 2). In contrast, IONPs functionalised with OA and PEG solution (PEG-OA-IONPs) were found to be colloidally stable for months without forming any precipitate (see Fig. 2). The online PEG functionalisation process, achieved by injecting into well-mixed reacting liquid slugs, demonstrated significantly improvements in terms of operation time, ease of operation, and production rate, compared to the traditional post-workup batch process [14, 15]. This can be attributed to good mixing characteristics from the internal slug circulation in segmented flow reactors. In the present study, a production rate of 2.28 g/day was achieved using 1 mm tubing. However, the process is readily scalable through the use of tubing with larger internal diameters.

**Fig. 3:**
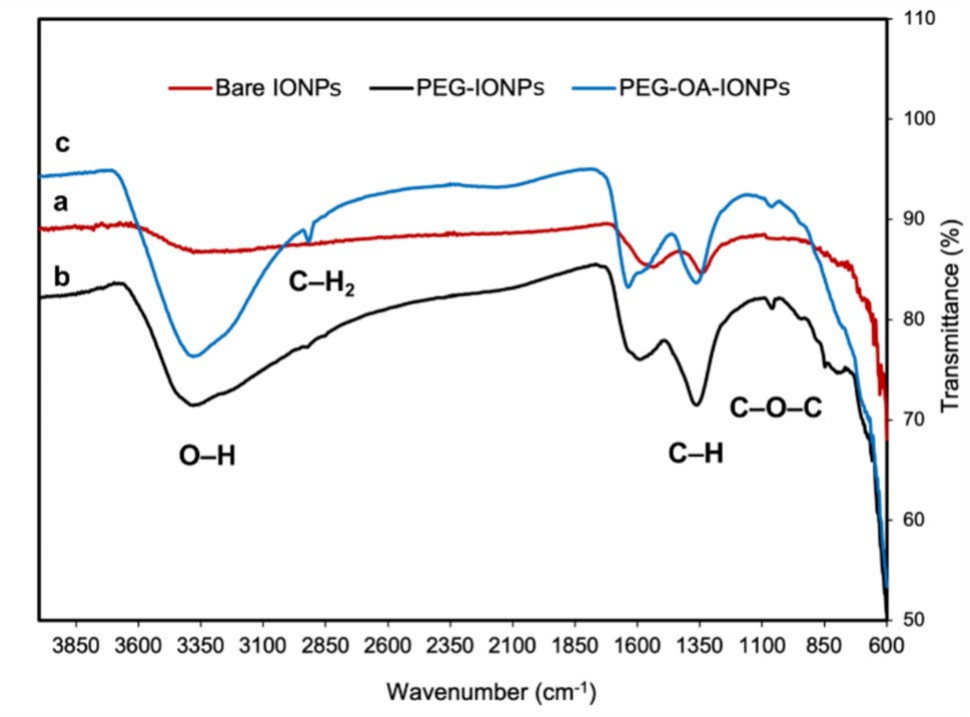
Fourier transform infrared spectroscopy (FT-IR) spectra of (**a**) Bare-IONPs, (**b**) PEG-IONPs, (**c**) PEG-OA-IONPs

The PEG functionalisation of IONPs was confirmed by FTIR analysis in the range of 600–4000 cm^−1^. The FTIR spectra of bare and PEG-functionalised IONPs are presented in Fig. 3. The plot displays distinct absorption peaks at specific wavenumbers, corresponding to the vibrations of particular functional groups. The spectra of bare and PEG-functionalised IONPs exhibit distinct differences, confirming the successful PEG functionalisation of the IONPs. The FTIR spectrum of PEG-functionalised IONPs shows peaks at 1360 cm⁻^1^, corresponding to the stretching vibration of the C–H group, and at 1070 cm⁻^1^, associated with the C–O–C stretching band. These findings confirm the presence of adsorbed PEG molecules on the surface of the IONPs [14, 26–28]. The hydroxyl groups of PEG generated a broad and intense peak in the FTIR spectrum, observed within the 3250–3500 cm⁻^1^ range [14, 27]. A shift in the PEG peak positions compared to pure PEG [14] was observed in the PEG-IONPs and PEG-OA-IONPs, which may indicate PEG binding to IONPs. A peak was observed at 2920 cm⁻^1^ only for PEG-OA-IONPs, which was indicative of the C–H_2_ stretching of oleic acid [21].

**Fig. 4:**
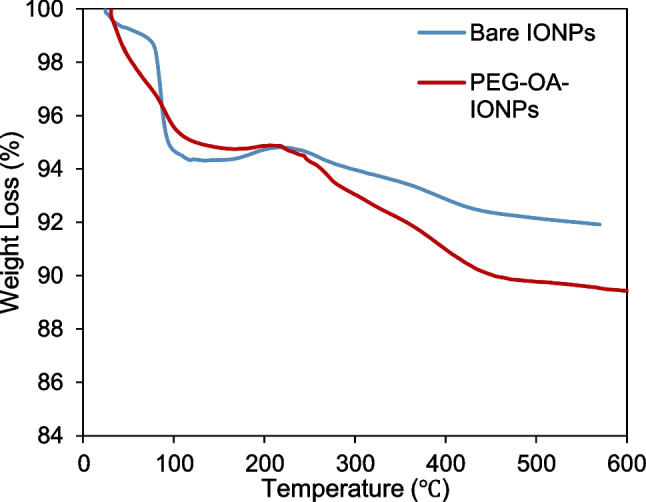
Thermogravimetric analysis curves of the bare-IONPs and PEG-OA-IONPs

Figure 4 presents the thermogravimetric analysis (TGA) of bare-IONPs and PEG-OA-IONPs. Both TGA curves show an initial weight loss up to 125 °C, attributed to the desorption of adsorbed water from the sample surface, followed by a significant weight loss above 200 °C. The total weight loss for bare-IONPs was 8%, while PEG-OA-IONPs exhibited a weight loss of 11.6%. The larger weight loss for PEG-OA-IONPs can be attributed to the decomposition and evaporation of PEG and oleic acid from the IONP surfaces, confirming the strong attachment of PEG molecules.

**Fig. 5:**
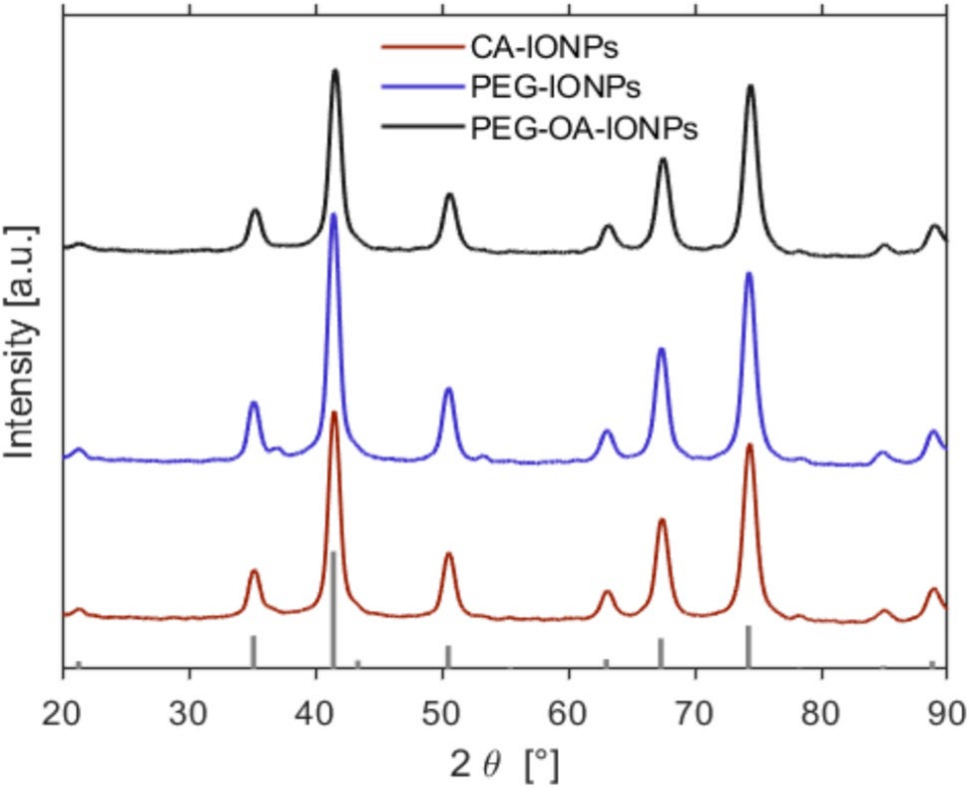
XRD patterns of functionalised IONPs that were synthesised in this work. The bars at the bottom show the peak positions and relative intensities corresponding to magnetite (PDF ref. 03–065–3107)

The XRD diffractograms of CA-IONPs, PEG-IONPs and PEG-OA-IONPs shown in Fig. 5 show that magnetite/maghemite was the only evident crystalline phase and there was no sign of any crystalline intermediate phases or byproducts. In addition, a clear supernatant was obtained (with negligible Fe content 0.02–0.05 mg/mL, measured by microwave plasma atomic emission spectroscopy) upon centrifuging the IONP samples, further confirming that no/negligible byproducts were formed.

### Biocompatibility/cytotoxicity study

**Fig. 6:**
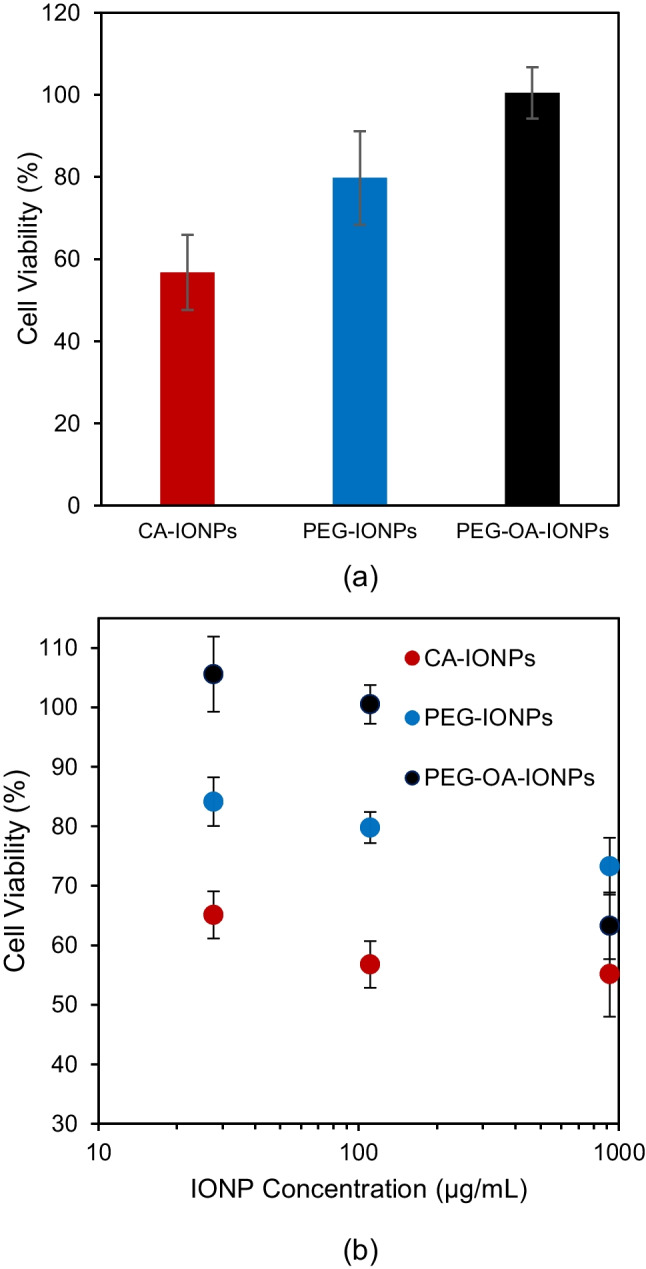
(**a**) Cell viability of IONPs after 24 h incubation at 110 µg/mL IONPs concentration. (**b**) Effect of IONP concentration on cell viability after 24 h

An increase in cell viability of IONPs with PEG functionalisation in comparison to CA-IONPs can be seen in Fig. 6a. Furthermore, as IONP concentration increased cell viability was found to decrease, as shown in Fig. 6b. Materials demonstrating cell viability exceeding 80% are generally considered biocompatible [29]. PEG functionalisation of the IONPs with the presence of oleic acid provided cell viability close to 100% and did not show any cytotoxic effect up to 110 $\mu$g/mL concentration, so it can be considered biocompatible at this concentration. However, with higher concentrations of the IONPs, a significant cytotoxic effect was observed for both PEG-functionalised IONPs.

### Magnetic properties of IONPs

**Fig. 7:**
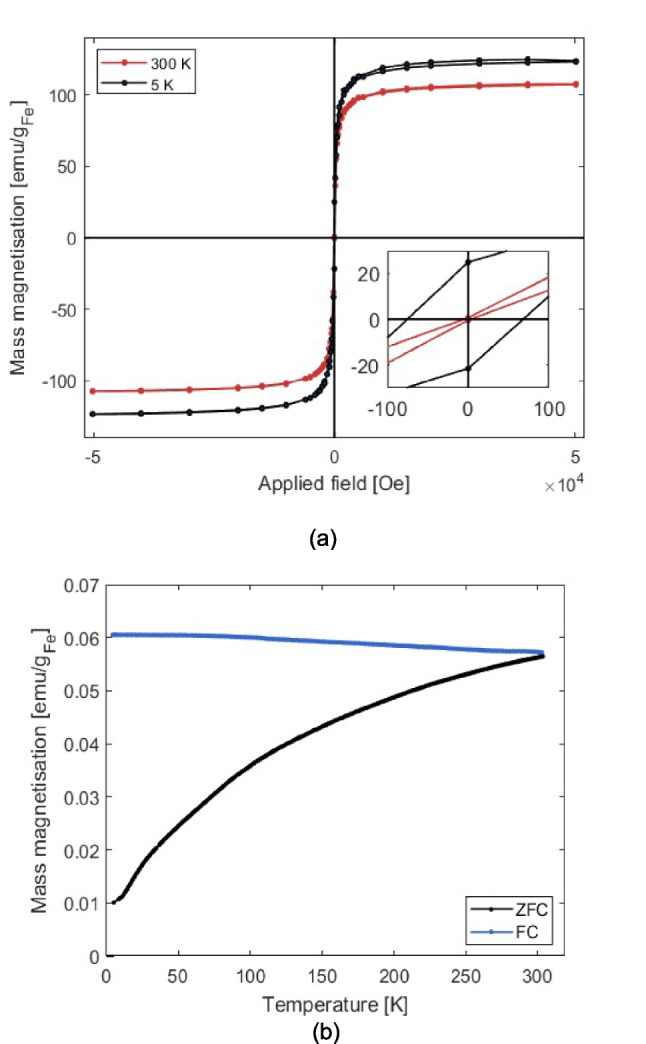
(**a**) Magnetic hysteresis curves for PEG-OA-IONPs; (**b**) Zero-field-cooling, ZFC, and field cooling, FC, curves of the PEG-OA-IONPs synthesised

Figure 7a shows the magnetisation curves of dried PEG-OA-IONPs. At 300 K, the curve is anhysteretic, indicating negligible coercivity and superparamagnetic behaviour, with a saturation mass magnetisation of 108.6 emu/g_Fe_; this is below the bulk magnetisation of 128 emu/g_Fe_ for magnetite [30] due to surface effects. The blocking temperature was investigated through zero-field cooled/field cooled (ZFC/FC) measurements, as shown in Fig. 7b, which indicated that the blocking temperature was above room temperature. This suggests that while the IONPs are superparamagnetic close to room temperature, an increase in particle size (> 16 nm) or cluster formation could exceed the superparamagnetic limit, leading to agglomeration due to magnetic attraction.

### Magnetic heating properties of PEG functionalised IONPs

**Fig. 8:**
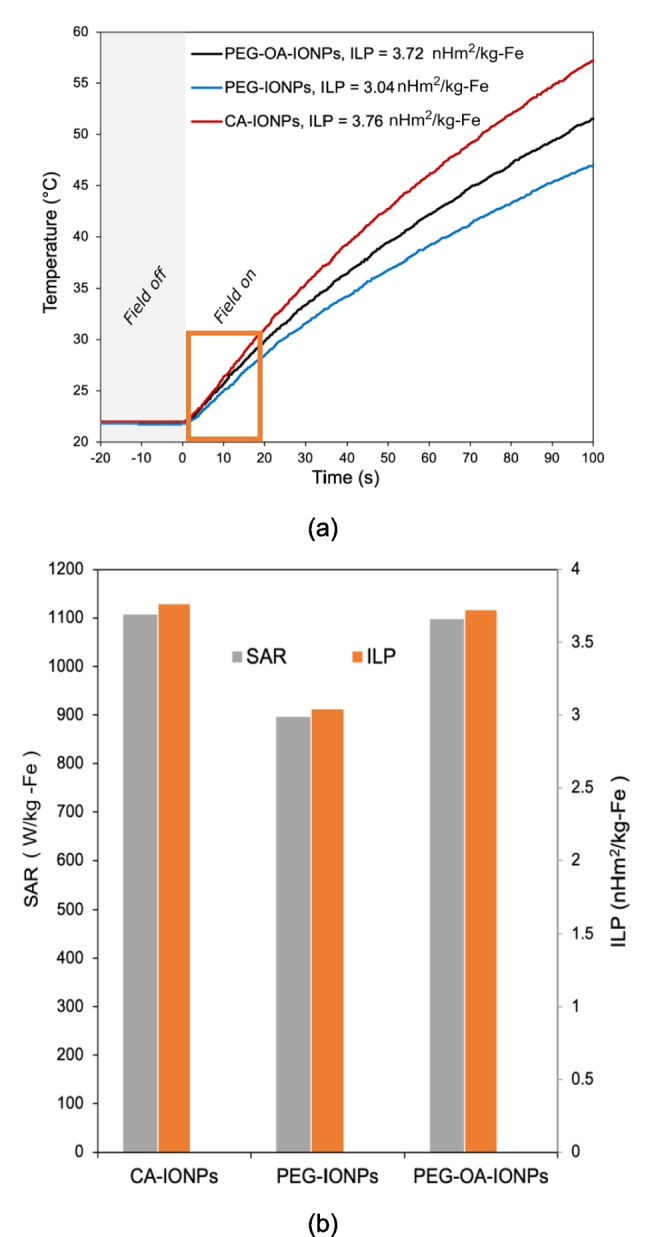
(**a**) Magnetic heating profiles, (**b**) Specific absorption rate (SAR) and Intrinsic loss power (ILP) values of IONPs with different functionalisation at a magnetic field strength of 23.87 kA/m and a frequency of 488 kHz

Figure 8a shows the magnetic heating profiles of the IONPs with different functionalisations at a magnetic field strength of 23.87 kA/m and a frequency of 488 kHz. The heating rates of CA-IONPs, PEG-IONPs, and PEG-OA-IONPs samples over the first 20 s did not vary significantly. The specific absorption rate (SAR) and intrinsic loss power (ILP) values [23] of the measured IONPs are presented in Fig. 8b. The results indicate that the PEG functionalisation of the IONPs, in the presence of oleic acid has a negligible impact on their heating performance. The ILP value of PEG-OA-IONPs remains essentially unchanged at 3.72 $\pm$ 0.39 nHm^2^ kg_Fe_^−1^ compared to the citrate stabilised IONPs without PEG functionalisation (3.76 $\pm$ 0.28 nHm^2^ kg_Fe_^−1^). It is important to highlight that IONPs functionalised with PEG without oleic acid (PEG-IONPs) were unstable in the long term, but still showed good heating performance (ILP 3.04 $\pm$ 0.24 nHm^2^ kg_Fe_^−1^). These results show that good biocompatibility by means of the PEG functionalisation of the IONPs was achieved, without compromising the magnetic properties of the nanoparticles, unlike previous reports [31].

### Photothermal assessment of functionalised IONPs

**Fig. 9:**
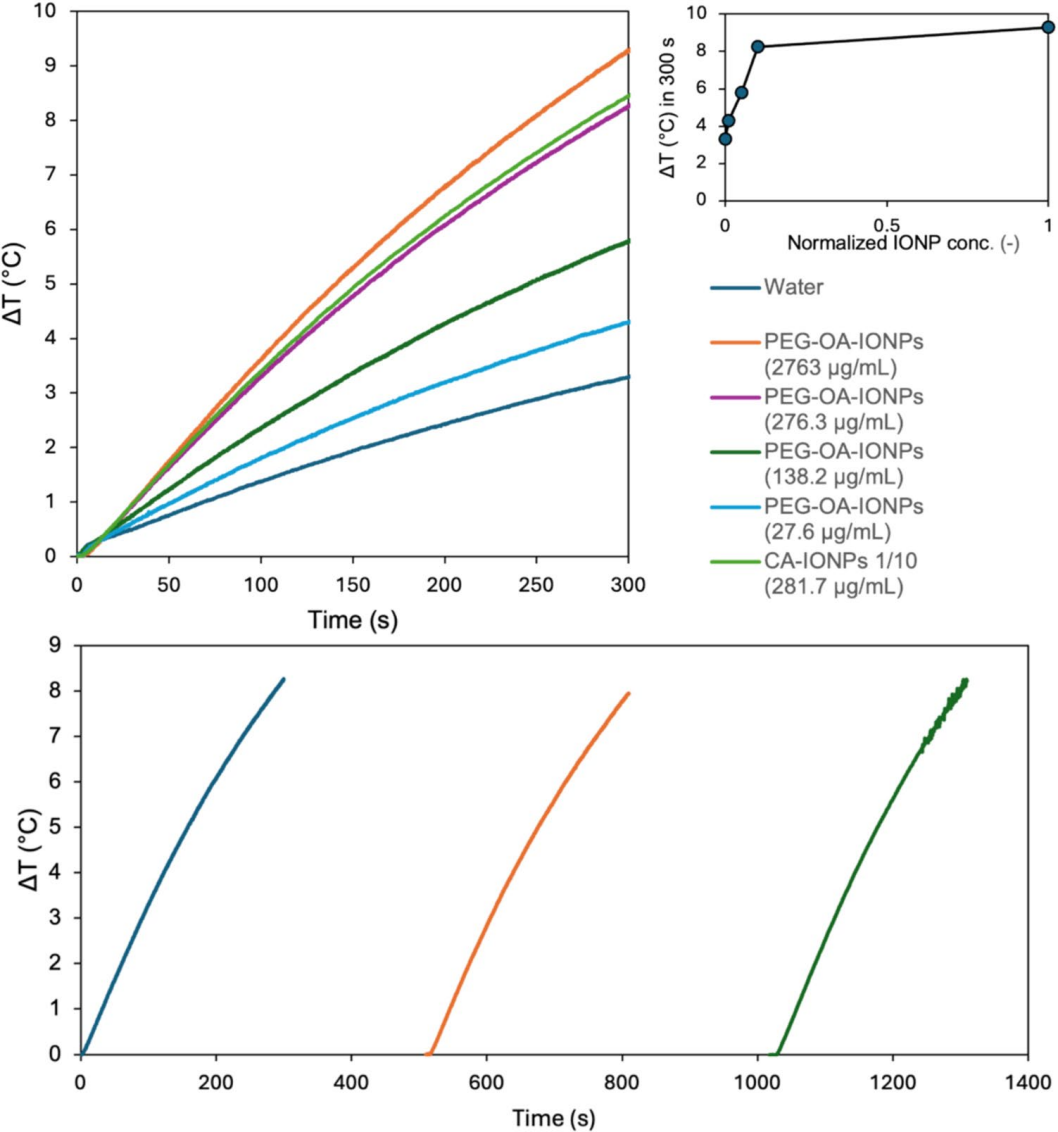
(Top) Photothermal response of PEG-OA-IONPs at different concentrations and photothermal response data for CA-IONPs at 281.7$\mu$g/mL. The inset shows the trend of the temperature increase as a function of normalised IONP concentration (sample IONP conc./IONP conc. of undiluted sample) for PEG-OA-IONPs. (Bottom) Assessment of PEG-OA-IONPs reusability for photothermal application under repeated irradiation sessions (at 276.3 $\mu$g/mL). All tests were performed at a wavelength of 810 nm at 420 mW IR-LED power

The photothermal performance of the PEG-OA-IONPs and CA-IONPs was evaluated by measuring the temperature increase of the nanoparticle suspension over time while under NIR irradiation. The photothermal response results, presented in Fig. 9 (top), show a clear photothermal effect of the nanoparticles. The maximum temperature increase within 5 min of irradiation (Fig. 9 (top, inset)) was found to be dependent on the nanoparticle concentration. For PEG-OA-IONPs, it was proportional to concentration at dilutions < 10% and approached a plateau with further concentration increase. This trend has been observed in previous work for polydopamine nanoparticles and was attributed to low penetration of light through the cuvette at high nanoparticle concentration [25]. Reproducibility was evaluated by multiple measurements of the photothermal response for a 1/10 diluted sample (276.3 $\mu$g/mL) and was found within ± 0.5 °C. The PEG-OA-IONPs were subjected to three cycles of photothermal heating and subsequent cooling, exhibiting consistent photothermal performance with minimal degradation due to thermal strain or photobleaching (see Fig. 9, bottom). These results highlight their potential reusability in photothermal applications.

While the temperature increase (or photothermal response) demonstrated here seems modest compared with previous reports [8, 32] it should be noted that comparison of photothermal response data is challenging due to significant differences in experimental procedures and setups, such as temperature probe placement, as reported in our previous work [25]. A sample of CA-IONPs was also measured at a 1/10 dilution, leading to an almost identical response with the PEG-OA-IONPs sample at the same dilution. This indicates that in our study good biocompatibility using the PEG functionalisation of the IONPs was achieved without compromising the photothermal properties of the IONP nanoparticles.

### Antifungal activity of PEG functionalised IONPs

**Fig. 10:**
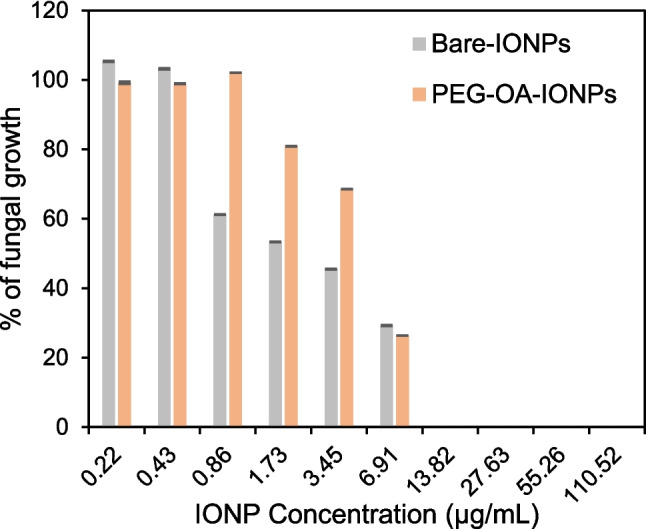
Fungal growth of *Trichophyton rubrum* as a function of the concentration of IONPs with and without PEG-OA functionalisation after 1 wk of incubation at 35 °C

Both IONPs without any functionalisation (bare-IONPs) and with PEG-OA functionalisation (PEG-OA-IONPs) showed antifungal activity against *Trichophyton rubrum* at concentrations at and above 1.73 $\mu$g/mL. This reflects numerous reports of the antifungal activity of nanoparticles composed of metals and metal oxides, including silver, gold, palladium, zinc oxide, copper oxide and iron oxide, against a range of fungal species [12, 33]. The mechanisms of action, although still to be definitely established, are thought to be via damage to the fungal cell wall, inhibition of ergosterol synthesis, gene regulation, interaction with reproductive structures and hyphae, and production of reactive oxygen species [33]. In our experiments, we found the minimum fungicidal concentration (MFC), i.e. the lowest concentration of an antifungal agent that kills 99.9% of a fungal inoculum to be ~ 14 $\mu$g/mL for both bare-IONPs and PEG-OA-IONPs solutions (Fig. 10). At ~ 7 $\mu$g/mL, PEG-OA-IONPs were found to be slightly more effective against *Trichophyton rubrum* compared to bare-IONPs. However, with lowering concentrations (up to 0.86 $\mu$g/mL) bare-IONPs were found to be more effective than PEG-OA-IONPs. This could be due to PEG-OA moieties reducing interactions between the nanoparticles and fungal cells. At lower concentrations (< 0.86 $\mu$g/mL), both bare-IONPs and PEG-OA-IONPs were ineffective at inhibiting fungal growth. Overall, our experiments show that the online PEG functionalisation was successful in synthesising IONPs which are capable of demonstrating antifungal activity at concentrations where they exhibit good biocompatibility.

## Conclusion

Continuous synthesis of magnetic IONPs followed by rapid online PEG functionalisation was performed using a three-phase droplet injection reactor platform. Colloidally stable PEG-functionalised IONPs were synthesised in the presence of oleic acid. A significant increase in biocompatibility was observed with PEG functionalisation of IONPs. Furthermore, the functionalised nanoparticles maintained key magnetic properties-saturation magnetisation, magnetic heating performance (ILP and SAR), and photothermal heating performance-crucial for their application in therapeutic contexts. This integrated synthesis and functionalisation platform not only reduces processing time and complexity but also ensures consistent quality and scalability at g/day rates, paving the way for industrial adoption. The results of this study underscore the potential of PEG-functionalised IONPs as a multifunctional therapeutic tool for cancer treatment, photothermal applications, and antifungal formulations.

## Data Availability

Data will be made available upon request.
